# Identifying the interaction between skin temperature, maintained thermal comfort, and conduit artery shear rate through limb passive heating

**DOI:** 10.14814/phy2.70923

**Published:** 2026-06-10

**Authors:** Eva‐Lotte Schabbehard, Stephanie Nessler, Justin S. Lawley

**Affiliations:** ^1^ Department of Sport Science, Division of Performance Physiology and Prevention University of Innsbruck Innsbruck Austria

**Keywords:** brachial artery, limb passive heating, local thermal comfort, retrograde shear rate, shear rate, superficial femoral artery, thermal balance, water perfused sleeve

## Abstract

Cardiovascular disease remains the leading cause of mortality. One reason is vascular dysfunction leading to pathological shear rate pattern. Limb passive heating (LPH) is a non‐pharmacological intervention, improving vascular health by enhancing shear rate. As LPH is intended for long‐term application, it is essential to identify a tolerable heating intensity that effectively reduces retrograde and increases antegrade shear rate without causing thermal discomfort. This study aimed to determine an LPH intensity that balances effective shear rate levels with thermal comfort. In 15 healthy participants (26 ± 3 years), LPH was applied to the right arm and leg targeting skin temperatures of resting baseline, 35°C and 40°C, while the contralateral limbs were maintained at 30°C as controls. After 10 min of steady‐state heating, brachial and superficial femoral retro‐, and antegrade shear rates, and local thermal comfort were assessed. Brachial artery: antegrade shear rate (35°C, *p* = 0.006; 40°C, *p* < 0.0001) increased, retrograde shear rate (*p* = 0.006) reduced at 40°C, and local arm thermal comfort was 8 ± 1. Femoral artery: antegrade shear rate (35°C, *p* < 0.0001; 40°C, *p* < 0.0001) and retrograde shear rate (35°C, *p* = 0.001; 40°C, *p* < 0.0001) altered significantly compared to controls, with local leg thermal comfort at 9 ± 1. These findings suggest through statistical interpolation, on average a skin temperature of approximately 38°C is perceived as thermally comfortable during acute LPH.

## 1 INTRODUCTION

Cardiovascular disease (CVD) is the leading cause of death worldwide (World Health Organization, 2022), with the number of deaths increasing by 18.7% from 2010 to 2020 (Tsao et al., 2022). Two underlying features of this challenging disease are vascular stiffness (Hooglugt et al., 2022) and endothelial dyfunction (Hooglugt et al., 2022; Poznyak et al., 2022). Vascular stiffness is caused by remodeling of the large major arteries and resistance vessels (Boutouyrie et al., 2021; Hooglugt et al., 2022; Tesauro et al., 2017) that ultimately contributes to arterial hypertension. Endothelial dysfunction is characterized by, in part, impaired nitric oxide mediated vasomotor reactivity, vascular permeability, and inflammation, which leads to a weakening in organ perfusion and the development of atherosclerosis.

Mechanisms contributing to arterial stiffness (Lacolley et al., 2020) and endothelial dysfunction (Lacolley et al., 2020) are multifactorial, but low levels of shear stress on the vascular wall are a common feature (Lv et al., 2024; Thosar et al., 2012; Zhou et al., 2023). Previous data have shown that engaging in lifelong aerobic exercise, which causes periods of high vascular shear rates (Amin et al., 2022), diminishes the age‐related reduction in vascular stiffness and endothelial dysfunction (Sarma et al., 2020). Moreover, experimental evidence has attenuated the rise in vascular shear rate associated with exercise training and prevented the associated vascular remodeling and improvements in flow‐mediated endothelial function (Tinken et al., 2010). Therefore, exercise and periods of high shear stress are a non‐pharmacological approach to prevent and/or reverse arterial stiffness and endothelial dysfunction. Yet many individuals fail to adopt a consistent exercise regime due to constraints such as limited time availability, reduced motivation, and self‐efficacy. Furthermore, aging and the onset of disease reduce cardiac and pulmonary reserve contributing to exercise intolerance (Chen et al., 2022).

An alternative non‐pharmacological approach that has been proposed to prevent or treat arterial stiffness and endothelial dysfunction is whole body or limb passive heat (LPH) exposure (Brunt & Minson, 2021). Whole‐body passive heating (WBPH) has a positive effect on the vasculature comparable to exercise (Amin et al., 2021) through an increase in vascular shear stress (Langille, 1996) and the addition of heat shock proteins that protect the endothelial cells against stress and denaturation (Hu et al., 2022; Zininga et al., 2018). Indeed, acute (Didier et al., 2022) and chronic (Brunt, Howard, et al., 2016) WBPH therapy has been shown to improve macro‐ and microvascular function. Although WBPH has demonstrated great potential as a strategy for controlling vascular disease, large‐scale implementation faces several challenges. Access to appropriate facilities is limited, and these facilities are costly to maintain due to the high energy demands required for sustaining elevated water temperatures. Additionally, unfit individuals and those with CVD are at increased risk of falling due to wet surfaces and experience thermal intolerance (Cramer et al., 2022), which restricts the overall exposure time. An alternative strategy may be the application of LPH. LPH exposure dilates the cutaneous circulation (Minson et al., 2001) and, depending on temperature and duration, the skeletal muscle circulation (Richey et al., 2024), which reduces downstream resistance and, as a consequence, increases blood flow and shear stress through the conduit and resistance arteries perfusing that limb. Hence, LPH may improve conduit and resistance arteries through heightened shear rate (Langille, 1996) and improved endothelial function in the macro‐ (Carter et al., 2014; Naylor et al., 2011) and microvasculature (Cheng et al., 2021; Green et al., 2010). This improvement in endothelial function also seems possible without a corresponding elevation in core temperature (Coombs et al., 2021).

To date, the intensity of WBPH and LPH has mostly been “intense” (typically water temperature ~42°) and while causing high shear rates, also causes a feeling of thermal discomfort that limits exposure times (typically ~30 min). Conversely, like exercise training, there may be similar and additional benefits to a lower intensity of LPH and shear rate over prolonged periods of time (i.e., several hours). Indeed, a recent study documented a 28 mmHg reduction in overnight systolic blood pressure in patients with autonomic failure by sleeping (8 h) on a moderately warm (38°C) water‐perfused heating pad (Okamoto et al., 2021). At present, there is no experimental evidence defining individual preferences for divergent heating strategies (high vs. low intensity) or whether these approaches produce comparable effects. This gap largely reflects limited understanding of the interaction between skin temperature, thermal comfort, and local shear rates during LPH, making it difficult to prescribe an optimal prolonged heating stimulus. Whereas exercise tolerance is primarily determined by the power–duration relationship and perceived exertion (Clark et al., 2019; Marcora, 2009; Marcora et al., 2009), tolerance to passive heat exposure is governed mainly by thermal perception and subjective discomfort, driven by cutaneous thermoreceptor activation in response to changes in skin temperature (Nagashima et al., 2018). Based on the idea of implementing long‐term LPH therapy to improve vascular stiffness and function, we aim to characterize the relationship between skin temperature, thermal comfort and shear rate as one of the primary factors responsible to improving vascular health. In doing so, these data would provide a general guideline as to a target skin temperature that is generally perceived as thermally comfortable during LPH. We hypothesized that progressive heating of the skin on the arm and leg would cause a linear increase in thermal discomfort and a corresponding linear reduction in retrograde shear rate alongside an increase in antegrade shear rate. Based on pilot data and data from Okamoto et al. (2021), it was hypothesized that a skin temperature ~38°C would be tolerable with a thermal comfort of feeling warm but fairly comfortable and a substantial reduction of retrograde shear rate with a sufficient increase in antegrade shear rate in healthy young individuals to serve as a therapeutic stimulus (Crandall & Wilson, 2015; Havenith, 2001; Havenith & Fiala, 2015; Marcora, 2008).

## 2 METHODS

### 2.1 Participants

Fifteen healthy participants (male = 6, female = 9) volunteered in this study. Inclusion criteria: all participants were regularly engaged in physical activity (self‐reported 9 ± 3 h of exercise per week). Exclusion criteria were smokers and individuals with cardiac, pulmonary, or metabolic diseases. Male (age, 25 ± 3 years; height, 181.42 ± 6.61 cm; weight, 73.17 ± 6.87 kg; BMI, 22.24 ± 1.92 kg/m^2^) and female (age, 27 ± 3 years; height, 168.27 ± 4.41 cm; weight, 61.15 ± 5.9 kg; BMI, 21.61 ± 2.11 kg/m^2^) participants were right‐handed, and the intervention was performed on their dominant side. Before all trials, participants refrained from intense exercise, the consumption of caffeine and alcohol for 24 h. Female participants were measured in the early follicular phase of their cycle. Informed written consent was obtained after each participant was given a verbal and written explanation of the experimental protocol and fully understood the possible risks involved in taking part in the study. The study protocol was approved by the ethics committee at University of Innsbruck and followed the principles from the Declaration of Helsinki.

### 2.2 Experimental protocol

**FIGURE 1:**
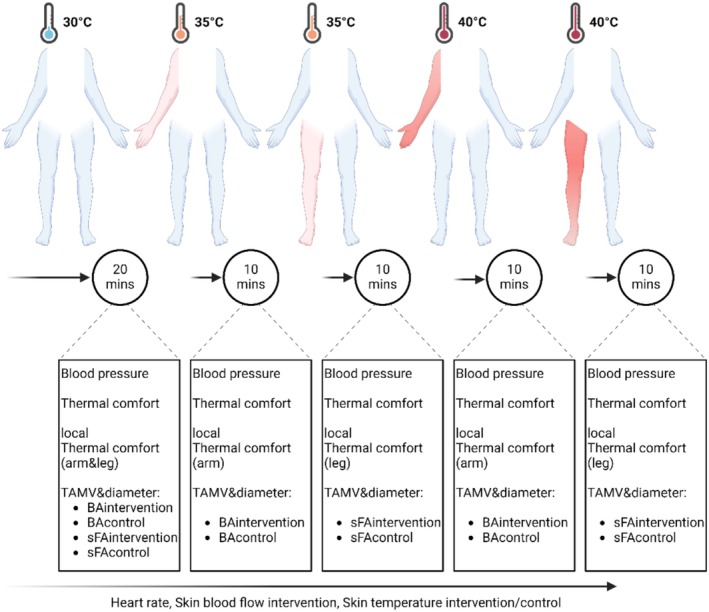
Schematic representation of the experimental protocol. The initial 20‐min period represents the resting phase prior to baseline measurements, followed by four consecutive 10‐min steady‐state heating phases at different local temperatures (35°C arm, 35°C leg, 40°C arm, and 40°C leg). After the resting phase (baseline) and each heating phase, measurements were performed as represented in the boxes below the timeline, including blood pressure, thermal comfort (whole‐body and local), and vascular parameters (TAMV and diameter for brachial artery and superficial femoral artery, intervention and control). Time durations indicate steady‐state conditions for each thermal state. Break varied because skin temperature was allowed to return to baseline before initiating the next heating period.

All measurements were completed in a quiet, environmentally controlled physiology laboratory (ambient temperature, 21.68°C ± 1.6°C; humidity, 36.67 ± 7.04%, barometric pressure, 955.61 ± 7.62 mmHg; time of day, 8 o'clock). After receiving ethical approval, body weight (Kern DS 150 k1; Kern & Sohn, Germany) and height were measured, participants were situated in a semi‐recumbent position (45°) on a hospital bed, equipped for measurements and all extremities were controlled at 30°C skin temperature with water perfused sleeves. After 20 min the baseline hemodynamic data were measured. Thereafter, the intervention arm was heated to a target skin temperature of 35°C using a custom‐made water perfused tube‐lined sleeve. After skin temperature had reached a steady state all measurements, including hemodynamic variables and thermal comfort were performed in the intervention arm followed by the counter lateral arm as control after 10 min steady state heating. Heart rate, skin temperature, and skin blood flow were measured continuously throughout the whole protocol. First thermal comfort and thermal sensation were always asked after 10 min steady state skin temperature. To exclude any overlapping reflex or central responses due to cutaneous heating between the interventions, and to ensure the same heating duration for both heating intensities the next measurement was conducted after the hemodynamics and the arm skin temperature had returned to baseline skin temperature of 30°C. Subsequently, the leg was heated to a targeted skin temperature of 35°C skin temperature with a custom‐made water perfused tube‐lined legging. Once leg skin temperature reached a steady state for 10 min all measurements were performed in the intervention leg followed by the control leg. The leg skin temperature was subsequently allowed to return to baseline skin temperature, and the exact same protocol was repeated with a target arm and leg skin temperature of 40°C (Figure 1). Both the water perfused tube‐lined sleeve and legging were regulated by a circulating bath equipped with a digital temperature controller (PolyScience, USA).

### 2.3 Experimental measurements

#### 2.3.1 Thermal regulatory variables

Intervention forearm and leg skin temperature and skin blood flow were continuously obtained via an integrated thermistor and a laser‐Doppler flowmeter (Moor Instruments, Devon, UK). A purpose‐built probe holder (PH2) was affixed to the ventral aspect of the forearm (mid‐region) with great care to avoid contact with disruptive factors as skin hair or veins. A large‐area optic probe (LP7A/T; 2 mm ring of collecting fibers) emitting laser light (wavelength 785 nm; intensity ~1 mW) was used to measure cutaneous red blood cell flux, expressed in perfusion units. The probe was inserted into the holder until gentle contact with the skin was achieved, with care taken to avoid cutaneous compression. The probe cable was secured to the skin using a strain relief loop to ensure consistent positioning and minimize movement artifacts throughout the experimental protocol. Control forearm and leg skin temperature were measured with T‐type IEC insulated copper constant thermocouples (OMEGATM, USA) and TC‐20000 device (Sable Systems; USA). Whole‐body thermal comfort, arm local thermal comfort, and leg local thermal comfort were enquired with McGinnis 13‐point scale (1 = so cold I am helpless to 7 = comfortable to 13 = so hot I am sick and nauseate). Thermal sensation, arm local thermal sensation, and leg local thermal sensation were asked via a thermal sensation scale (−3 = cold to 0 = neutral to +3 = hot) (Deng et al., 2017).

#### 2.3.2 Cardiovascular variables

Heart rate was continuously recorded using a three‐lead electrocardiogram (Tram‐rac, Solar 8000M GE, Marquette, USA). Blood pressure was taken intermittently from the left arm by electrosphygmomanometry (Tango, M2, SunTechMedical Instruments Inc., USA) with a microphone placed over the left brachial artery to detect Korotkoff sounds.

#### 2.3.3 Ultrasonography

Brachial and superficial femoral artery time averaged mean velocity (TAMV) was measured using a 9‐MHz linear‐array Doppler probe (iE33; Philips, The Netherlands) by continuous duplex vascular sonography (iE33; Philips, The Netherlands). Arterial diameter was imaged using two‐dimensional B mode over 60 s and measured offline during diastole in triplicate by the same investigator using calipers. Anatomical landmarks visible during B‐mode measurements of diameter were noted alongside a skin marker that was used to ensure consistent probe placement between baseline and all subsequent recordings. Brachial artery and superficial femoral artery TAMV was recorded at an insonation angle of 60° for 60 s and imported into LabChart via a Doppler audio converter (Penn State, Hershey, Pennsylvania) (Herr et al., 2010) and the sample volume encompassing the entire vessel lumen. Antegrade and retrograde TAMV data were captured in separate channels within LabChart. All measurements were conducted by the same investigator and basic ultrasound settings including depth, gain, power, dynamic range, and sample volume were kept constant for each participant over time.

### 2.4 Data acquisition and analysis

All continuous measurements were sampled at 250 Hz using a Powerlab (Powerlab; AD Instruments, Oxford, UK) and extracted via an offline data acquisition system (LabChart 8; AD Instruments, Oxford, UK) as averages over 60 s. Mean arterial pressure (mmHg) was calculated via the following equation: mean arterial pressure = 2/3 diastolic blood pressure + 1/3 systolic blood pressure. Brachial artery and superficial femoral artery blood flow (mL·min^−1^) were calculated with the following equation: blood flow = {TAMV · [π · (arterial diameter/2)^2^]} · 60. Antegrade or retrograde blood flow of the brachial and superficial femoral artery were calculated with this equation: antegrade or retrograde blood flow = {antegrade or retrograde TAMV · [π · (arterial diameter/2)^2^]} · 60. To calculate brachial artery and superficial femoral artery shear rate (s^−1^) this equation was used: shear rate = 4 · (TAMV/diameter). The calculation of antegrade and retrograde shear rate was antegrade or retrograde shear rate = 4 · (antegrade or retrograde TAMV/diameter).

### 2.5 Statistical analysis

A repeated‐measurement ANOVA with Holm‐Šídák multiple comparisons test was conducted to assess differences between baseline and both heating conditions for heart rate, mean arterial pressure, intervention arm and leg skin blood flow, thermal comfort, arm thermal comfort, leg thermal comfort, thermal sensation, arm thermal sensation, and leg thermal sensation. Additionally, a mixed‐effects analysis with Holm‐Šídák multiple comparisons test was performed to evaluate differences among the skin temperatures of 30°C, 35°C, and 40°C on the intervention side, as well as to compare the intervention and control side at each skin temperature level. This analysis included the following variables: skin temperature, TAMV, antegrade TAMV, retrograde TAMV diameter, blood flow, antegrade blood flow, retrograde blood flow, shear rate, antegrade shear rate, and retrograde shear rate across all extremities, including the brachial and superficial femoral arteries. All variables (except thermal comfort and sensation) were averaged over a 60‐s period, measured always after 10 min steady state skin temperature conditions. Pearson's correlation coefficient *r* was used to describe the strength of the relationship between arm or leg skin temperature, local thermal comfort, and brachial artery or superficial femoral artery retrograde shear rate and antegrade shear rate of the intervention extremity. A two‐tailed *p* value was calculated. All values are expressed as mean ± standard deviation with a statistical significance set at *p* ≤ 0.05 for post hoc analysis and *ANOVA p* ≤ 0.05 for repeated‐measurement ANOVA. Prism 9 (GraphPad Software Inc., La Jolla, CA) was used for statistical analysis including the Shapiro–Wilk normality test.

## 3 RESULTS

### 3.1 Influence of forearm and leg LPH on cardiovascular responses and thermal comfort and sensation

During forearm LPH, heart rate (ANOVA *p* = 0.21) and mean arterial pressure (ANOVA *p* = 0.223) remained unchanged, whereas leg LPH at 35°C caused a small but significant increase in heart rate (*p* = 0.023) without altering mean arterial pressure (ANOVA *p* = 0.132). Local thermal comfort and sensation increased in the heated limb during both forearm and leg LPH from baseline to 35°C (forearm: *p* = 0.0001; leg: *p* = 0.0002) and to 40°C (forearm: *p* < 0.0001; leg: p = 0.0001). Whole body thermal comfort and sensation also rose, with forearm LPH showing higher comfort at 40°C (*p* = 0.032) and sensation at 35°C and 40°C (*p* = 0.005), while leg LPH increased whole body comfort (baseline to 35°C: *p* = 0.013; baseline to 40°C: *p* = 0.001) and sensation (baseline to 35°C: p = 0.0002; baseline to 40°C: *p* < 0.0001). Skin blood flow of the heated limb increased significantly for forearm (35°C: *p* = 0.006; 40°C: p < 0.0001) and leg LPH (35°C and 40°C: *p* < 0.0001) (see Tables S1–S3).

### 3.2 Influence of forearm and leg LPH at 35°C and 40°C on the intervention limb and compared to the contralateral control limb

#### 3.2.1 Limb passive heating—Forearm

**FIGURE 2:**
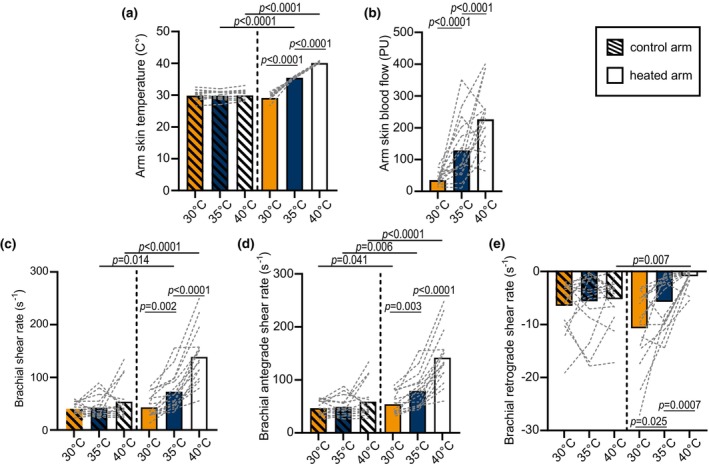
Comparison of control arm (dashed bars) and the heated arm at three different skin temperature levels: 30°C (orange), 35°C (blue), and 40°C (white). The illustrated variables include skin temperature (a), skin blood flow (b), brachial artery shear rate (c), antegrade shear rate (d), and retrograde shear rate (e). *p* Values above the graphs indicate comparisons between the control and intervention arm, while *p*‐values above the heated arm graphs denote comparisons of measurements in the heated arm at 30°C versus 35°C and 30°C versus 40°C.

Forearm LPH at targeted skin temperatures of 35°C and 40°C significantly increased skin temperature (*p* < 0.0001) and skin blood flow in the intervention limb compared to baseline, with both temperatures higher than the control limb (Figure 2a). At 35°C, TAMV in the brachial artery was elevated versus baseline (*p* = 0.001) and control (*p* = 0.007) without diameter changes (baseline: *p* = 0.952; control: *p* = 0.669), leading to higher brachial artery blood flow (baseline: *p* = 0.002; control: *p* = 0.003) via increased antegrade flow (baseline: *p* = 0.003; control: *p* = 0.002), while retrograde flow decreased (became less negative) versus baseline (*p* = 0.022) but was not different from the control limb (*p* = 0.709). Mean and antegrade shear rates rose (baseline: *p* = 0.002 and *p* = 0.003; control: *p* = 0.014 and *p* = 0.006), whereas retrograde shear rate decreased relative to baseline (*p* = 0.025) but not control (*p* = 0.959). At 40°C, TAMV, brachial artery blood flow, and antegrade flow all increased significantly versus baseline (*p* < 0.0001) and control (TAMV: *p* < 0.0001; blood flow: *p* = 0.0001; antegrade flow: *p* < 0.0001), also retrograde flow decreased versus baseline (*p* = 0.0003) and versus control (*p* = 0.022). Shear rates were similarly affected, with mean, antegrade, and retrograde shear rates all significantly higher or lower (baseline: *p* < 0.0001, *p* < 0.0001, *p* = 0.001; control: *p* < 0.0001, *p* < 0.0001, *p* = 0.007), and diameter remained unchanged (baseline: *p* = 0.117; control: *p* = 0.231) (Figure 2, Tables S1 and S3).

#### 3.2.2 Limb passive heating—Leg

**FIGURE 3:**
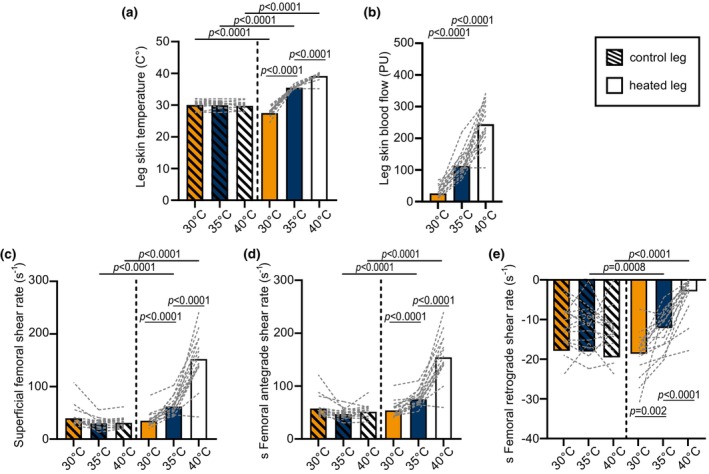
Comparison of control leg (dashed bars) and the heated leg at three different skin temperature levels: 30°C (orange), 35°C (blue), and 40°C (white). The illustrated variables include skin temperature (a), skin blood flow (b), superficial femoral artery shear rate (c), antegrade shear rate (d), and retrograde shear rate (e). *p* Values above the graphs indicate comparisons between the control and intervention leg, while *p* values above the heated leg graphs denote comparisons of measurements in the heated leg at 30°C versus 35°C and 30°C versus 40°C.

Leg LPH at targeted skin temperatures of 35°C and 40°C significantly increased skin temperature (*p* < 0.0001) and skin blood flow in the intervention limb compared to baseline, with both temperatures higher than the control limb (Figure 3a). At 35°C, TAMV in the femoral artery increased versus baseline and control (*p* < 0.0001), without diameter changes (baseline: *p* = 0.759; control: *p* = 0.359), resulting in elevated femoral artery blood flow (baseline and control: *p* < 0.0001) via increased antegrade flow (baseline: *p* = 0.0004; control: *p* < 0.0001), while retrograde flow decreased versus baseline (*p* = 0.043) but was not different from control (*p* = 0.166). Mean and antegrade shear rates were higher (*p* < 0.0001), whereas retrograde shear rate increased (became less negative) versus baseline (*p* = 0.002) and increased versus control (*p* = 0.001). At 40°C, TAMV, femoral artery blood flow, and antegrade flow all increased versus baseline and control (*p* < 0.0001), while retrograde flow decreased versus baseline (*p* < 0.0001) and versus control (*p* = 0.001). Shear rates were similarly affected, with mean, antegrade, and retrograde shear rates all significantly higher or lower (*p* < 0.0001), and femoral artery diameter remained unchanged (baseline: *p* = 0.769; control: *p* = 0.564) (Figure 3, Tables S2 and S3).

### 3.3 Balance of local thermal comfort and retrograde shear rate during limb passive heating

**FIGURE 4:**
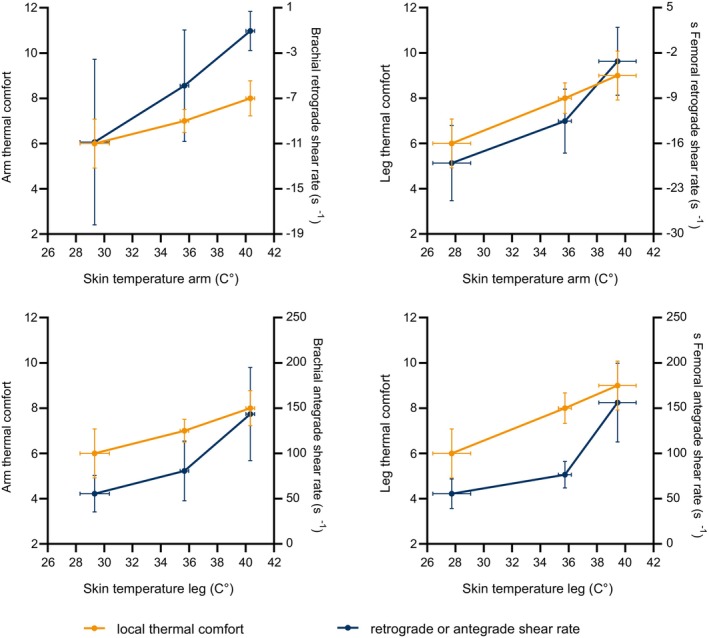
The orange line presents the mean local thermal comfort for both the arm and leg, demonstrating a strong linear correlation with skin temperature. The blue line depicts retrograde and antegrade shear rate for the brachial and superficial femoral arteries. While thermal comfort shows a linear relationship with skin temperature, the relationship between skin temperature and shear rate is generally non‐linear, with the possible exception of retrograde shear rate in the brachial artery. The balance for prolonged local limb passive heating occurs at a local thermal comfort level of 8 (“warm but fairly comfortable”), corresponding to a skin temperature of 40.32°C ± 0.31°C for the arm and 35.73°C ± 0.46°C for the leg. Shear rate values are reported at these respective temperatures (i.e., the conditions corresponding to a thermal comfort level of 8). In the brachial artery (40°C condition), retrograde shear rate is −1.05 ± 1.73 s^−1^ (a), while antegrade shear rate is 143.46 ± 51.48 s^−1^ (c). In the superficial femoral artery (35°C condition), retrograde shear rate is −12.55 ± 4.94 s^−1^ (b), and antegrade shear rate is 79.5 ± 14.61 s^−1^ (d).

Strong correlations were observed between skin temperature and local thermal comfort as well as shear rates in both the arm and leg. In the arm, skin temperature correlated with thermal comfort (*r* = 0.774, *p* < 0.0001), retrograde shear rate (*r* = 0.635, p < 0.0001), and antegrade shear rate (*r* = 0.676, *p* < 0.0001). At a local thermal comfort of 8 (“warm but fairly comfortable”), arm skin temperature reached 40.32°C ± 0.31°C, decreasing retrograde shear rate from −10.87 ± 7.31 s^−1^ to −1.05 ± 1.73 s^−1^ and increasing antegrade shear rate from 55.51 ± 20.06 s^−1^ to 143.46 ± 51.48 s^−1^. In the leg, skin temperature similarly correlated with thermal comfort (*r* = 0.718, *p* < 0.0001), retrograde shear rate (*r* = 0.775, *p* < 0.0001), and antegrade shear rate (*r* = 0.757, *p* < 0.0001). At a thermal comfort of 8, leg skin temperature reached 35.73°C ± 0.46°C, reducing retrograde shear rate from −19.04 ± 5.8 s^−1^ to −3.3 ± 5.26 s^−1^ and increasing antegrade shear rate from 55.53 ± 16.26 s^−1^ to 156.08 ± 43.51 s^−1^ (Figure 4).

## 4 DISCUSSION

The main findings of this present study were that LPH of the skin to 40°C causes a 605% and 856% increase in skin blood flow to the arm and leg, respectively. This decrease in cutaneous vascular resistance caused an important upstream decrease (less negative) in retrograde shear rate in the brachial and superficial femoral artery alongside an increase in antegrade shear rate in both arteries. In contrast to our hypothesis, this followed a bi‐phasic pattern increasing substantially after skin temperature reached ~36°C. Overall responses were greater with 40°C skin temperature than at 35°C skin temperature. On average, LPH caused a linear positive relationship between skin temperature and local thermal comfort. While whole‐body thermal comfort and local thermal comfort stayed tolerable during both LPH intensities in the arm, local leg thermal comfort increased to an uncomfortable level with a leg skin temperature of 40°C. This suggests that long duration LPH used as a therapy for endothelial function (Brunt, Eymann, et al., 2016; Brunt, Howard, et al., 2016; Brunt & Minson, 2021; Carter et al., 2014; Cheng et al., 2021) should be individually adapted to the upper and lower extremity and lies in between ~36°C and 40°C skin temperature.

### 4.1 Sweet spot skin temperature between shear rate and local thermal comfort

A strong positive linear correlation was observed between both the leg or arm skin temperature and the perception of local thermal comfort (see Figure 4). Retrograde shear rate in the arm exhibited a linear relationship with skin temperature (see Figure 4a). However, upon closer examination, the brachial antegrade shear rate (see Figure 4c), along with both antegrade and retrograde shear rates in the femoral artery (see Figure 4d,b), demonstrated a nonlinear biphasic relationship. As the balance between stimulus (skin temperature) and response (shear rate) for prolonged LPH likely occurs at a local thermal comfort level of ~8 (“warm but fairly comfortable”), this corresponds to a skin temperature of ~40.32°C ± 0.31°C for the arm and ~35.73°C ± 0.46°C for the leg.

Retrograde shear rates demonstrated a greater reduction at 35°C skin temperature in the brachial artery than in the superficial femoral artery. This difference between limbs likely reflects different changes in microvascular resistance between the arm and the leg. Retrograde shear rate in an upstream conduit artery is affected by changes in downstream skin blood flow (Simmons et al., 2011) and thus cutaneous microvascular resistance. Therefore, an obvious expiration would be that cutaneous microvascular resistance was lower in the arm than in the leg at 35°C. However, we observed similar changes in skin blood flow in the heated arm and leg at 35°C skin temperature. These data suggest that the difference in retrograde shear rate between limbs may be related to a greater reduction in skeletal muscle vascular resistance in the arm compared to the leg. Indeed, several studies have shown that LPH of the skin increases muscle temperature, decreases skeletal muscle vascular resistance and alters blood flow in the skeletal muscle and upstream conduit arteries (Chiesa et al., 2016; Heinonen et al., 2011; Watanabe et al., 2024). As the muscle‐skin ratio is greater in the leg than in the arm, it is possible that the attenuated reduction (i.e., less negative) in retrograde shear rate in the leg was due to a smaller increase in leg muscle temperature at 35°C due to thermal inertia. This would result in an attenuated reduction in skeletal muscle vasodilation and vascular resistance in the leg compared to the arms. Antegrade shear rate on the other hand exhibited in both the brachial and superficial femoral arteries a similar biphasic relationship with skin temperature. Since antegrade shear rate is determined by the product of antegrade TAMV and the inverse of arterial diameter, and no significant differences in diameter were observed in either artery, the primary determinant of antegrade shear rate in this study was antegrade TAMV. The bi‐phasic relationship observed for antegrade shear rate in the femoral artery aligns with the retrograde shear rate pattern, as both share the same intensity of response to skin temperatures of 35°C and 40°C. This can be explained by the fact that retrograde shear rate and antegrade shear rate respond inversely to changes in pressure difference and vascular resistance (Widmaier et al., 2004).

Thermal comfort reflects a sensation that limits the optimal duration and intensity of LPH exposure (Bud Craig, 2018). While whole‐body thermal comfort remained ≤8, which indicates a comfortable thermal tolerance, this must be interpreted with caution, especially as the targeted LPH therapy should heat all four extremities simultaneously. In such cases, whole‐body thermal comfort might increase. On average, LPH induced a linear positive relationship between skin temperature and local thermal comfort, though local thermal comfort responded differently in the arm and leg. Specifically, while the arm reached a “warm but fairly comfortable” score of 8 at 40°C skin temperature, the leg was rated as “uncomfortably warm” at the same temperature. This discrepancy may be due to the leg's greater skin surface area compared to the arm. Although, to the best of our knowledge, no research has compared skin sensitivity between the arm and leg during LPH, local thermal comfort should be considered independently for each limb based on this study results.

Local thermal comfort governs the maximum intensity of long duration LPH, while ineffective, too low shear rate dictates the minimum intensity. The optimum LPH intensity, reflected in skin temperature, corresponds to the point at which retrograde shear rate is reduced, antegrade shear rate is efficiently high, and skin temperature is rated tolerable, leading to reduced resistance, enhanced mechanotransduction, and elevated nitric oxide levels. In the arm, a thermal comfort score of 8, corresponding to a skin temperature of approximately 40°C, resulted in almost no retrograde shear rate and a substantial increase in antegrade shear rate. In contrast, in the leg, subjective local thermal comfort limits skin temperature and the point at which retrograde shear rate is completely reduced. Assuming the observed linear relationship between leg thermal comfort and skin temperature holds true, a local thermal comfort score of 8 corresponds to a skin temperature of around 38°C (via statistical interpolation), with a retrograde shear rate of −8 s^−1^, representing a 36% decrease compared to baseline but a 181% increase in antegrade shear rate.

### 4.2 Comparison of limb passive heating with exercise and whole‐body passive heating

Exercise is well‐documented in preventing or delaying CVD through its multifaceted effects on the vasculature (Amin et al., 2021, 2022; Chen et al., 2022; Dibben et al., 2023; Kim et al., 2014; Mason et al., 2020; Pagan et al., 2018; Tinken et al., 2010). Similarly, WBPH, whether through immersion in hot water (Amin et al., 2021) resting in a dry sauna (Leach et al., 2024), or heating via a water perfused suit (Hoekstra et al., 2021), has demonstrated efficacy in preventing or delaying CVD by improving endothelial function (Bailey et al., 2016; Brunt, Eymann, et al., 2016; Brunt, Howard, et al., 2016; Brunt & Minson, 2021; Carter et al., 2014; Cheng et al., 2021; Didier et al., 2022; Green et al., 2010). While WBPH typically increases both skin, muscle, and core temperature (Hoekstra et al., 2021), LPH increases skin and muscle temperature (Hoekstra et al., 2021). Interestingly, while the current study observed a general correlation between skin temperature and conduit artery shear rate with LPH, the decrease in cutaneous vascular conductance does not explain, in its entirety, the increase in conduit artery blood flow with WBPH. For example, Hoekstra et al. (2021) observed that femoral blood flow increased by ~250 mL·min^−1^ by passively heating the legs to 38°C for 45 min, yet WBPH (+∆Trec ~1.5°C) elevated femoral blood flow by ~1100 mL·min^−1^. This was despite a similar change in both skin and deep muscle tissue temperature. These data and others (Koch Esteves et al., 2021), highlight the independent effect of a rise in core temperature on the physiological responses to passive heating, that heat‐activated thermosensitive mechanism also contribute to limb perfusion (Koch Esteves et al., 2021) and the major limitation of LPH. Our data demonstrate that LPH elicits comparable effects on conduit artery shear rates that are 80%–96% of those induced by exercise and WBPH (Amin et al., 2021, 2022; Hoekstra et al., 2021). Our measurements reveal a 420.55% increase in femoral artery shear rate relative to baseline, driven by a significant reduction in retrograde shear rate. Hoekstra et al. (2021) reported femoral artery blood flow of 1713 mL·min^−1^ during 90 min of WBPH and 943 mL·min^−1^ during LPH, while our study observed 423 mL·min^−1^ after only 10 min of LPH. Nevertheless, in most scientific studies, WBPH generally increases whole body thermal comfort to an uncomfortable score within 40–90 min (Amin et al., 2021; Hoekstra et al., 2021; Leach et al., 2024) limiting exposure time, whereas during LPH whole body thermal comfort is generally unaffected (Hoekstra et al., 2021) or as in the current study raised mildly, while local thermal comfort can be maintained at comfortable perceptions. This data implies that in theory, LPH may be favorable for the perception of thermal stress and applied for prolonged (several hours) treatment periods. Indeed, Gibson et al. (2023) recently performed 3 h of unilateral leg heating (50°C circulating water, skin (~40°C) and muscle (~38°C) temperature) and observed favorable increases in skeletal muscle gene transcription.

Despite these benefits, most studies limit therapy duration to 30–45 min (Cheng et al., 2021; Green et al., 2010; Naylor et al., 2011) due to high‐intensity protocols (e.g., water temperature ~42°C), which induce thermal discomfort and limit practical application. Both WBPH and high‐intensity LPH create high shear rates but also result in discomfort, typically restricting exposure to about 30 min. Conversely, similar to exercise, lower‐intensity LPH may yield comparable or even additional benefits if applied over prolonged durations (e.g., several hours). For instance, sleeping for 8 h on a moderately warm (38°C) water‐perfused heating pad reduced overnight systolic blood pressure by 28 mmHg in patients with autonomic failure (Okamoto et al., 2021), demonstrating the potential of extended moderate heating. Our findings based on interpolation between our measurement points indicate that LPH at 38°C would achieve the desired macrovascular effects (increase in shear rate) while maintaining thermal comfort. The stable thermal comfort suggests this stimulus could be applied for extended periods, potentially yielding greater long‐term benefits. Furthermore, the limitations of exercise (e.g., compliance) and WBPH (e.g., logistical challenges, slipping risks, and high‐water temperatures unsuitable for prolonged heating) underscore the advantages of LPH. In summary, WBPH induces a higher thermal discomfort rating, whereas LPH, despite triggering less microvascular perfusion initially, produces sustained vascular responses over time and can be tolerated for extended heating durations.

Atherosclerosis, a macrovascular disease, may benefit from heat therapy interventions (Benincasa et al., 2022; Brunt & Minson, 2021; Langille, 1996). Through graded manipulations in skin temperature, we have shown that isolated LPH of either the arm or leg can significantly increase skin blood flow, decrease vascular resistance, and nearly eliminate retrograde shear rate. Furthermore, our results indicate a 300% increase in anterograde shear rate in upstream conduit arteries. This evidence highlights LPH as a targeted and tolerable therapy over a long‐time duration with the potential to improve vascular function.

### 4.3 Limitations

During the experimental protocol core temperature was not measured. Hoekstra et al. (2021) measured no change in core temperature with LPH for 60 min (~37°C) (Hoekstra et al., 2021). This suggests that LPH affects only skin temperature in contrast to WBPH (Amin et al., 2021). Yet while other studies have observed a slight increase in core temperature with LPH of both legs for ~30 min (Carter et al., 2014; Cheng et al., 2021), they measured no changes in heart rate and mean arterial pressure. Given the short heating duration (10 min) and the absence of changes in heart rate and mean arterial pressure, an increase in core temperature was not expected. Accordingly, a limitation of the present study is both the brief duration and the unilateral application of peripheral heating. While this approach was deliberately employed to isolate limb‐specific interactions between skin temperature, thermal comfort and shear rates, it limits the generalizability of any therapeutic potential that would require prolong application of LPH over several weeks/months. Indeed, a similar limitation is that the current study only measured thermal comfort during isolated limb heating. To improve vascular health, it is expected that multiple limbs will be heated simultaneously, thus future research is needed to ascertain if there is an additive or synergistic effect of multi‐limb heating on thermal comfort. Moreover, many research studies have observed a progressive increase in limb perfusion with LPH (Cheng et al., 2021; Heinonen et al., 2011). The current approach aimed to isolate changes in antegrade and retrograde shear that could be expected from a change in cutaneous resistance. Thus a short time window where skin temperature and cutaneous conductance is markedly elevated yet deep tissue temperature remains relatively unchanged (Hoekstra et al., 2021) was chosen. Future research is needed to ascertain the overall changes in shear rates and thermal comfort during multi extremity passive heating. Another limitation is the acute nature of LPH to identify the “balanced” relationship between skin temperature, thermal comfort and vascular shear rate. The 10‐min heating duration reflects an acute exposure and may not fully capture responses during prolonged heat therapy interventions. Previous data has shown that femoral blood flow progressively increases overtime (60 min) with the application of a 50°C water perfused suit, suggesting that both hemodynamic and perceptual responses may evolve with longer exposure. If thermal comfort follows a similar pattern over time (i.e., quickly progressing to uncomfortably hot) with milder thermal stimulus as used in the current study is worthy of future study. An additional limitation of the present study is that the experimental protocol was conducted as a single trial without repeated measures or assessment of day‐to‐day reproducibility, limiting the ability to fully establish the reliability and consistency of the observed responses. This is particularly relevant given the modest magnitude of some changes in shear rate, which may be more susceptible to biological variability and measurement error. However, this limitation was partially mitigated by the inclusion of the contralateral limb as a simultaneous within‐subject control, allowing real‐time comparison and helping to account for systemic influences and intraindividual variability. Finally, only young healthy individuals participated in the study. While young individuals should aim to prevent vascular disease, the application of LPH is aimed at reversing age‐related declines in vascular health. Thus, it is important to translate these data to older individuals and perform long‐term studies on the effectiveness of LPH in reversing vascular stiffness and improving vascular function.

## 5 CONCLUSION

These findings demonstrate that local skin temperatures which are perceived as comfortable can produce meaningful increases in antegrade shear rate and reductions in retrograde shear rate (increase in absolute values) in both the arm and leg, effectively shifting the flow profile toward a more favorable pattern. While individual variability exists, through statistical interpolation, our data suggest that a skin temperature ~38°C is an effective starting point to develop prolonged low intensity heating paradigms in subsequent follow up studies.

## AUTHOR CONTRIBUTIONS

**Eva‐Lotte Schabbehard:** Conceptualization; data curation; formal analysis; investigation; methodology; project administration. **Justin S. Lawley:** Conceptualization; data curation; formal analysis; funding acquisition; investigation; methodology; project administration; supervision. **Stephanie Nessler:** Project administration.

## FUNDING INFORMATION

None.

## CONFLICT OF INTEREST STATEMENT

No conflicts of interest, financial or otherwise, are declared by the authors.

## ETHICS STATEMENT

It was paid by the University of Innsbruck. The ethical approval number is ethical approval number: 34/2018.

## Supporting information


Tables S1–S3.
