# Supplementary material for: Identifying the interaction between skin temperature, maintained thermal comfort, and conduit artery shear rate through limb passive heating
Source: Physiol Rep. 2026 Jun 10;14(11):e70923. doi: 10.14814/phy2.70923 (PMC13254236; doi:10.14814/phy2.70923)
Supplement: Supplementary file 1 — Tables S1–S3. [file PHY2-14-e70923-s001.docx]

**SUPPLEMENTARY MATERIAL**

**TABLES**

**Table S1.** Data of three skin temperature levels (30°C, 35°C, and 40°C) in the intervention arm. Time-controlled variables include heart rate, mean arterial pressure, whole-body thermal comfort, local arm thermal comfort, whole-body thermal sensation, local arm thermal sensation, and skin blood flow in the intervention arm. Additionally, variables controlled for both time and side – comparing the intervention arm to the control arm maintained at 30°C skin temperature – include skin temperature, time-average mean velocity (TAMV), antegrade TAMV, retrograde TAMV, diameter, blood flow, antegrade and retrograde blood flow, shear rate, and antegrade and retrograde shear rate.

| Local passive heating on the arm | | | | | | |  |
| --- | --- | --- | --- | --- | --- | --- | --- |
|  | **30°C** | | **35°C** | | **40°C** | |  |
| Heart rate (bpm) | 58 ± 9 | | 56 ± 8 | | 58 ± 7 | |  |
| Mean arterial pressure (mmHg) | 83 ± 7 | | 84 ± 8 | | 84 ± 8 | |  |
| Whole-body thermal comfort | 6 ± 1 | | 6 ± 1 | | 7 ± 1***** | |  |
| Local thermal comfort | 6 ± 1 | | 7 ± 1***** | | 8 ± 1***** | |  |
| Whole-body thermal sensation | -1.06 ± 0.72 | | -0.4 ± 0.74***** | | 0.47 ± 1.09***** | |  |
| Local thermal sensation | -1.06 ± 0.72 | | 1.01 ± 0.73***** | | 2.04 ± 0.79***** | |  |
| Skin blood flow (PU) | 37.9 ± 20.38 | | 131.4 ± 97.01***** | | 229.3 ± 103.2***** | |  |
|  | **control** | **heated** | **control** | **heated** | **control** | **heated** | |
| Skin temperature (°C) | 30.15 ± 1.51 | 29.32 ± 1.03 | 30.14 ± 1.31^†^ | 35.67 ± 0.3***** | 30.31 ± 1.45^†^ | 40.32 ± 0.31***** | |
| TAMV (cm·s^-1^) | 3.99 ± 1.24 | 4.33 ± 2.08 | 4.13 ± 1.7^†^ | 7.24 ± 3.53***** | 5.56 ± 3.84^†^ | 14.5 ± 5.56***** | |
| Antegrade TAMV (cm·s^-1^) | 4.68 ± 1.4 | 5.4 ± 1.9 | 4.75 ± 1.45^†^ | 7.91 ± 3.33***** | 6.11 ± 3.69^†^ | 14.96 ± 5.48***** | |
| Retrograde TAMV (cm·s^-1^) | -0.63 ± 0.41 | -1.03 ± 0.66 | -0.61 ± 0.54 | -0.55 ± 0.46***** | -0.51 ± -0.42^†^ | -0.11 ± 0.18***** | |
| Diameter (cm) | 0.39 ± 0.06 | 0.39 ± 0.06 | 0.39 ± 0.06 | 0.39 ± 0.05 | 0.4 ± 0.05 | 0.42 ± 0.04 | |
| Blood Flow (ml∙min^-1^) | 30.64 ± 16.96 | 32.62 ± 18.4 | 29.64 ± 15.94^†^ | 55.35 ± 32.8***** | 45.10 ± 39.55^†^ | 121.6 ± 55.31***** | |
| Antegrade blood flow (ml∙min^-1^) | 35.44 ± 17.84 | 40.30 ± 17.80 | 33.89 ± 15.28^†^ | 60.36 ± 32.49***** | 49.06 ± 38.83^†^ | 125.9 ± 56.07***** | |
| Retrograde blood flow (ml∙min^-1^) | -4.38 ± 2.55 | -7.34 ± 4.54 | -4.21 ± 3.57 | -3.77 ± 3.38***** | -3.71 ± 3.15^†^ | -0.9 ± 1.63***** | |
| Shear rate (s^-1^) | 40.85 ± 10.62 | 44.38 ± 21.24 | 43.82 ± 20.49^†^ | 73.66 ± 34.85***** | 54.97 ± 34.37^†^ | 139.4 ± 52.7***** | |
| Antegrade shear rate (s^-1^) | 48.28 ± 13.57^†^ | 55.51 ± 20.06 | 50.48 ± 18.25^†^ | 80.6 ± 32.94***** | 60.68 ± 32.87^†^ | 143.5 ± 51.48***** | |
| Retrograde shear rate (s^-1^) | -6.65 ± 4.81 | -10.87 ± 7.31 | -5.79 ± 5.97 | -5.88 ± 4.93***** | -5.35 ± 4.34^†^ | -1.05 ± 1.73***** | |

Data is presented as mean ± standard deviation. *****, *p*-values < 0.05 for time-control (heated arm 30°C versus 35°C and 30°C versus 40°C); †, *p*-values < 0.05 for side-control (heated arm versus control arm for 30°C, 35°C and 40°C).

**Table S2.** Comparison of three skin temperature levels (30°C, 35°C, and 40°C) in the intervention leg. Time-controlled variables include heart rate, mean arterial pressure, whole-body thermal comfort, local leg thermal comfort, whole-body thermal sensation, local leg thermal sensation, and skin blood flow in the intervention leg. Additionally, variables controlled for both time and side – comparing the intervention leg to the control leg maintained at 30°C skin temperature – include skin temperature, time-average mean velocity (TAMV), antegrade TAMV, retrograde TAMV, diameter, blood flow, antegrade and retrograde blood flow, shear rate, and antegrade and retrograde shear rate.

| Local passive heating on the leg | | | | | | |  |
| --- | --- | --- | --- | --- | --- | --- | --- |
|  | **30°C** | | **35°C** | | **40°C** | |  |
| Heart rate (bpm) | 58 ± 8 | | 56 ± 7***** | | 60 ± 9 | |  |
| Mean arterial pressure (mmHg) | 83 ± 7 | | 86 ± 9 | | 85 ± 9 | |  |
| Whole-body thermal comfort | 6 ± 1 | | 7 ± 1***** | | 8 ± 1***** | |  |
| Local thermal comfort | 6 ± 1 | | 8 ± 1***** | | 9 ± 1***** | |  |
| Whole-body thermal sensation | -1.06 ± 0.72 | | 0.41 ± 0.69***** | | 1.28 ± 1.04***** | |  |
| Local thermal sensation | -1.06 ± 0.72 | | 1.31 ± 0.62***** | | 2.44 ± 0.86***** | |  |
| Skin blood flow (PU) | 28.91 ± 17.69 | | 115.4 ± 38.65***** | | 247.5 ± 68.95***** | |  |
|  | **control** | **heated** | **control** | **heated** | **control** | **heated** | |
| Skin temperature (°C) | 30.34 ± 1.33^†^ | 27.81 ± 1.35 | 30.22 ± 1.12^†^ | 35.74 ± 0.48***** | 29.99 ± 1.2^†^ | 39.41 ± 1.35***** | |
| TAMV (cm·s^-1^) | 6.11 ± 2.61 | 5.42 ± 1.81 | 4.6 ± 1.39^†^ | 9.76 ± 2.34***** | 4.78 ± 1.37^†^ | 23.3 ± 6.05***** | |
| Antegrade TAMV (cm·s^-1^) | 8.83 ± 2.59 | 8.33 ± 1.73 | 7.21 ± 1.48^†^ | 11.86 ± 2.21***** | 7.87 ± 1.7^†^ | 23.88 ± 5.26***** | |
| Retrograde TAMV (cm·s^-1^) | -2.75 ± 0.76 | -2.88 ± 0.78 | -2.73 ± 0.75^†^ | -1.99 ± 0.88***** | -2.95 ± 0.8^†^ | -0.56 ± 0.92***** | |
| Diameter (cm) | 0.62 ± 0.07 | 0.61 ± 0.06 | 0.6 ± 0.07 | 0.62 ± 0.06 | 0.6 ± 0.06 | 0.62 ± 0.07 | |
| Blood Flow (ml∙min^-1^) | 107.3 ± 43.67 | 94.18 ± 24.86 | 79.17 ± 36.73^†^ | 179.9 ± 53.08***** | 81.78 ± 34.37^†^ | 423.6 ± 119.4***** | |
| Antegrade blood flow (ml∙min^-1^) | 155.5 ± 51.31 | 145.9 ± 29.5 | 125 ± 47.97^†^ | 220.7 ± 65.76***** | 134.8 ± 49.22^†^ | 436.2 ± 109.6***** | |
| Retrograde blood flow (ml∙min^-1^) | -48.44 ± 15.69 | -51.24 ± 18.78 | -46.57 ± 15.99 | -38.44 ± 23.04***** | -50.49 ± 18.58^†^ | -12.23 ± 21.46***** | |
| Shear rate (s^-1^) | 40.98 ± 21.3 | 36.31 ± 15.34 | 31.04 ± 10.03^†^ | 63.18 ± 16.53***** | 32.33 ± 10.69^†^ | 152.7 ± 47.91***** | |
| Antegrade shear rate (s^-1^) | 59.05 ± 21.7 | 55.53 ± 16.26 | 48.48 ± 9.78^†^ | 76.5 ± 14.61***** | 53.16 ± 13.29^†^ | 156.1 ± 43.51***** | |
| Retrograde shear rate (s^-1^) | -18.31 ± 5.76 | -19.04 ± 5.8 | -18.45 ± 5.54^†^ | -12.55 ± 4.94***** | -19.93 ± 5.6^†^ | -3.3 ± 5.26***** | |

Data is presented as mean ± standard deviation. *****, *p*-values < 0.05 for time-control (heated leg 30°C versus 35°C and 30°C versus 40°C); †, *p*-values < 0.05 for side-control (heated leg versus control leg for 30°C, 35°C and 40°C).

**Table S3.** *P*-values for arm and leg local passive heating measurements are presented. Time-controlled variables contain heart rate, mean arterial pressure, whole-body thermal comfort, local thermal comfort, whole-body thermal sensation, local thermal sensation, and skin blood flow in the intervention extremity. These were calculated using a repeated-measurement ANOVA with Holm-Šídák multiple comparisons test. Post-hoc comparisons were conducted between 30°C versus 35°C and 30°C versus 40°C measurements. Additionally, mixed-effects analysis with Holm-Šídák multiple comparisons tests was performed for time- and side-controlled variables, including skin temperature, time-average mean velocity (TAMV), antegrade TAMV, retrograde TAMV, diameter, blood flow, antegrade and retrograde blood flow, shear rate, and antegrade and retrograde shear rate. Time-controlled post-hoc comparisons assessed differences between 30°C versus 35°C and 30°C versus 40°C measurements for the heated extremity. Side-controlled post-hoc tests compared heated versus control extremities at 30°C, 35°C and 40°C measurements. The significance level was set at *p* = 0.05.

| Local passive heating intervention – Post-hoc and ANOVA *p*-values | | | | | | |
| --- | --- | --- | --- | --- | --- | --- |
|  | **Arm and Brachial artery** | | | **Leg and superficial femoral artery** | | |
| Repeated- measurement ANOVA with Holm -Šídák multiple comparisons test | | | | | | |
|  | **30°C vs 35°C** | **30°C vs 40°C** | **ANOVA p-value** | **30°C vs 35°C** | **30°C vs 40°C** | **ANOVA p-value** |
| Heart rate (bpm) | 0.202 | 0.805 | 0.21 | 0.023 | 0.154 | 0.003 |
| Mean arterial pressure (mmHg) | 0.32 | 0.32 | 0.223 | 0.123 | 0.517 | 0.132 |
| Whole-body thermal comfort | 0.071 | 0.032 | 0.009 | 0.013 | 0.001 | 0.0006 |
| Local thermal comfort | 0.0001 | <0.0001 | <0.0001 | 0.0002 | 0.0001 | <0.0001 |
| Whole-body thermal sensation | 0.005 | 0.005 | 0.002 | 0.0002 | <0.0001 | <0.0001 |
| Local thermal sensation | <0.0001 | <0.0001 | <0.0001 | <0.0001 | <0.0001 | <0.0001 |
| Skin blood flow (PU) | 0.006 | <0.0001 | <0.0001 | <0.0001 | <0.0001 | <0.0001 |
|  |  |  |  |  |  |  |
| Mixed-effects analysis with Holm -Šídák multiple comparisons test | | | | | | |
| Heated extremity | **30°C vs 35°C** | **30°C vs 40°C** | **ANOVA p-value** | **30°C vs 35°C** | **30°C vs 40°C** | **ANOVA p-value** |
| Skin temperature (°C) | <0.0001 | <0.0001 | <0.0001 | <0.0001 | <0.0001 | <0.0001 |
| TAMV (cm·s^-1^) | 0.001 | <0.0001 | <0.0001 | <0.0001 | <0.0001 | <0.0001 |
| Antegrade TAMV (cm·s^-1^) | 0.002 | <0.0001 | <0.0001 | <0.0001 | <0.0001 | <0.0001 |
| Retrograde TAMV (cm·s^-1^) | 0.022 | 0.001 | <0.0001 | 0.002 | <0.0001 | <0.0001 |
| Diameter (cm) | 0.952 | 0.117 | 0.028 | 0.759 | 0.769 | 0.259 |
| Blood Flow (ml∙min^-1^) | 0.002 | <0.0001 | <0.0001 | <0.0001 | <0.0001 | <0.0001 |
| Antegrade blood flow (ml∙min^-1^) | 0.003 | <0.0001 | <0.0001 | 0.0004 | <0.0001 | <0.0001 |
| Retrograde blood flow (ml∙min^-1^) | 0.022 | 0.0003 | <0.0001 | 0.043 | <0.0001 | <0.0001 |
| Shear rate (s^-1^) | 0.002 | <0.0001 | <0.0001 | <0.0001 | <0.0001 | <0.0001 |
| Antegrade shear rate (s^-1^) | 0.003 | <0.0001 | <0.0001 | <0.0001 | <0.0001 | <0.0001 |
| Retrograde shear rate (s^-1^) | 0.025 | 0.001 | 0.0003 | 0.002 | <0.0001 | <0.0001 |
| Control versus heated extremity | **30°C vs 30°C** | **30°C vs 35°C** | **30°C vs 40°C** | **30°C vs 30°C** | **30°C vs 35°C** | **30°C vs 40°C** |
| Skin temperature (°C) | 0.068 | <0.0001 | <0.0001 | <0.0001 | <0.0001 | <0.0001 |
| TAMV (cm·s-1) | 0.485 | 0.007 | <0.0001 | 0.151 | <0.0001 | <0.0001 |
| Antegrade TAMV (cm·s-1) | 0.071 | 0.002 | <0.0001 | 0.261 | <0.0001 | <0.0001 |
| Retrograde TAMV (cm·s-1) | 0.068 | 0.704 | 0.009 | 0.489 | 0.003 | <0.0001 |
| Diameter (cm) | 0.958 | 0.669 | 0.231 | 0.866 | 0.359 | 0.564 |
| Blood Flow (ml∙min^-1^) | 0.66 | 0.003 | 0.0001 | 0.156 | <0.0001 | <0.0001 |
| Antegrade blood flow (ml∙min^-1^) | 0.217 | 0.002 | <0.0001 | 0.273 | <0.0001 | <0.0001 |
| Retrograde blood flow (ml∙min^-1^) | 0.054 | 0.709 | 0.022 | 0.462 | 0.166 | 0.001 |
| Shear rate (s^-1^) | 0.438 | 0.014 | <0.0001 | 0.158 | <0.0001 | <0.0001 |
| Antegrade shear rate (s^-1^) | 0.041 | 0.006 | <0.0001 | 0.267 | <0.0001 | <0.0001 |
| Retrograde shear rate (s^-1^) | 0.147 | 0.959 | 0.007 | 0.526 | 0.001 | <0.0001 |
